# The Cupping-glass in Retention of Urine

**Published:** 1848-05

**Authors:** 


*The Cupping-glass in Retention of Urine.*—M. Vandenbroeck, principal physician at Mons, informs the Society of Medecine at Anvers, that for more than twenty years he has replaced the use of the catheter in both sexes by the application of large cupping glasses to the superior and internal part of the thigh. In nine cases out of every twelve, the emission of urine, says he, was made at the end of some seconds. He does not assert that this has any effect in mechanical retention of urine.—*Nouvelliste Medical Belge.*

